# Where Sex Meets Gender: How Sex and Gender Come Together to Cause Sex Differences in Mental Illness

**DOI:** 10.3389/fpsyt.2022.856436

**Published:** 2022-06-28

**Authors:** Dorte M. Christiansen, Margaret M. McCarthy, Mary V. Seeman

**Affiliations:** ^1^Department of Psychology, National Centre for Psychotraumatology, University of Southern Denmark, Odense, Denmark; ^2^Department of Pharmacology, University of Maryland, Baltimore, MD, United States; ^3^Department of Psychiatry, University of Toronto, Toronto, ON, Canada

**Keywords:** sex differences, gender differences, mental health, diathesis-stress model, posttraumatic stress disorder, autism spectrum disorders, eating disorders

## Abstract

Sex differences are prevalent in multiple mental disorders. Internalizing disorders are more commonly diagnosed in women, whereas externalizing and neurodevelopmental disorders are more often diagnosed in men. Significant sex/gender differences are reported in prevalence, symptom profile, age of onset, comorbidities, functional impairment, prognosis, as well as in responses to various treatments. In this conceptual article, we discuss theories and empirical studies of sex- and gender-related influences in mental health, by focusing on three examples: autism spectrum disorder (ASD), acknowledged as a disorder whose roots are mainly biological; eating disorders, whose origins are considered to be mainly psychosocial, and posttraumatic stress disorder (PTSD), an environmentally caused disorder with both psychosocial and biological underpinnings. We examine the ways in which sex differences emerge, from conception through adulthood. We also examine how gender dichotomies in exposures, expectations, role assumptions, and cultural traditions impact the expression of our three selected mental illnesses. We are especially interested in how sex-based influences and gender-based influences interact with one another to affect mental illness. We suggest that sex and gender are multi-faceted and complex phenomena that result in variations, not only between men and women, but also within each sex and gender through alterations in genes, hormone levels, self-perceptions, trauma experiences, and interpersonal relationships. Finally, we propose a conceptual diatheses-stress model, depicting how sex and gender come together to result in multiple sex/gender differences across mental disorders. In our model, we categorize diatheses into several categories: biological, intrapersonal, interpersonal, and environmental. These diatheses interact with exposure to stressors, ranging from relatively minor to traumatic, which allows for the sometimes bidirectional influences of acute and long-term stress responses. Sex and gender are discussed at every level of the model, thereby providing a framework for understanding and predicting sex/gender differences in expression, prevalence and treatment response of mental disorders. We encourage more research into this important field of study.

## Introduction

This hypothesis and theory paper is based on observations of a substantial effect of sex and gender on the expression of many mental disorders. We consider “sex” and “gender” to be two different, though overlapping, concepts. Both refer to the designation of “male/masculine” or “female/feminine,” but research has traditionally addressed the two terms differently. Individual disciplines have used different methodologies, and sometimes different terminologies to study sex and gender. Sex is generally defined as the biological distinction between males and females based on sex chromosomes XX or XY, established at conception. The male/female distinction is identified visually, even prior to birth, by the structure of external genitalia, and is later confirmed by its consistency with internal reproductive organs.

In contrast to sex, gender signifies one's relationship to the subjective perception of oneself as male or female. Gender is grounded in biological sex and strongly influenced by genotype and the prenatal hormonal environment, yet it is also subject to the perception and expectations of others and affected by parental rearing practices, by environmental exposures, by the development of sexual preferences, by cultural pressures, and by the process of identity formation. Research on the influence of sex has used biological tools, and has often been based on animal models. Research on gender has used anthropological or sociological terminology and methodology, with observations often viewed through a feminist lens. Though often treated as separate constructs, the two (sex and gender) are inseparable and neither can be fully understood in the absence of the other.

The worldwide prevalence of some mental disorders is reported as significantly more prevalent in girls and women whereas other disorders are more often diagnosed in boys and men. As a general rule of thumb, from puberty onward, internalizing disorders (those during which negative emotions are subjectively experienced) are more commonly seen in women (1–3). This group of disorders includes, among others, disordered eating and disorders and posttraumatic stress disorder (PTSD) (1, 4–7), which are characterized by subjective feelings of low self-esteem and insecurity, a tendency to self-blame, and a habit of inhibiting the outward expression of anger (8). In contrast, externalizing disorders, where distress is outwardly expressed through impulsive or aggressive behavior, are more commonly reported in men (8). Externalizing disorders are characterized by behaviors rather than by emotions, and by impaired ability to communicate feelings (1, 4, 6, 7, 9). Another general distinction with respect to prevalence, is that disorders that first manifest in childhood are usually more prevalent in boys. This is true for autism spectrum disorders (ASD) (10, 11). The rates of mental disorders more commonly seen in women rise after puberty and come to surpass those of men. In the elderly, male/female differences in prevalence tend to wane (11). For some DSM-5 disorders, the extent of prevalence differences by sex/gender is substantial (please see Table 1).

**Table 1:** F:M sex differences across mental disorders.

**Diagnosis**	**OR**	**95% CI**	**References**
MDD	2.0^a^	1.9–2.2^*^	(12)
Binge-eating disorder	2.3	1.8–2.9^*^	(13)
Bulimia nervosa	3.5	2.6–4.7^*^	(13)
PTSD	2.0^a^	1.8–2.2^*^	(14)
GAD^d^	1.8	1.7–2.0^*^	(15)
ASD	4.3^b^	4.0–4.6	(16)
Schizophrenia	1.0^c^	0.9–1.2	(17)
Bipolar I disorder	0.9	0.7–1.1	(18)
ADHD	0.4^a^	0.4–0.5^*^	(19)
Alcohol use disorder	0.5^a^	0.5–0.5^*^	(20)
Drug use disorder	0.6^a^	0.5–0.6^*^	(21)
ASPD	0.3^a^	0.2–0.3	(22)
Agoraphobia	1.9^d^	1.5–2.4	(23)

*PR, F:M prevalence ratio; MDD, major depressive disorder; PTSD, posttraumatic stress disorder; FAD, generalized anxiety disorder; ASD, autism spectrum disorder; ADHD, attention deficit hyperactivity disorder; ASPD, antisocial personality disorder*.

a *Converted from source to represent odds ratio in women compared to men*.

b *Sex ratio for ASD caseness among 8-year-old children*.

c *Data are based on a Chinese population; other data are based on Western populations, mostly from the USA*.

d *Data based on DSM-5 only diagnosis; similar results were found for those meeting both DSM-5 and DSM-IV criteria*.

* *Sex difference significant at p < 0.05*.

Whereas, most research on sex/gender differences in mental disorders has focused on prevalence and symptom severity, there also exist differences in the nature of symptoms, in age of onset, in frequency of episode recurrence, in chronicity, comorbidities, precursors, triggers, functional impairment, and treatment response (24, 25). In some cases, sex/gender differences are found in the psychiatric comorbidities associated with a predominant mental disorder -e.g., aggressive behavior and substance use disorder in men, contrasting with eating disorders and depression in women (26–30). The purpose of this hypothesis and theory paper is to apply new interpretations of recent data to the role of sex and gender in mental illness. Our aim is to highlight how sex and gender coalesce to result in differences between men's and women's mental health, the focus of this special Frontiers issue. Our three selected diagnoses each represent different poles of the sex/gender continuum, with ASD being more commonly diagnosed in boys, PTSD presenting in both sexes, and eating disorders seen far more frequently in women. Another important distinction among the three is that ASD begins early in life; eating disorders usually start at adolescence, and PTSD in adulthood. Finally, these three disorders were selected because they are differently influenced by diatheses (predispositions) and stressors (triggers): ASD more by biological diatheses, eating disorders more by societal diatheses, and PTSD requiring, by definition, a traumatic stressor. These three disorders thus fit into different niches of the new theoretical framework that we will present at the end of this paper.

### Autism Spectrum Disorders

ASD is ~4 times more prevalent in boys than in girls (16, 31). The source of this marked difference remains unclear; its basis is likely to be multifactorial but is poorly understood. There is no doubt about the biological etiology of ASD—these disorders are highly heritable. But reasons for the sex ratio in prevalence is a mystery because few of the 100+ thus far identified risk genes reside on sex chromosomes. Baron-Cohen has proposed the “extreme male brain theory” of ASD, attributing sex differences to testosterone levels (32). This is plausible because multiple lines of evidence suggest dysregulated steroidogenesis (33, 34), but testosterone levels are unlikely to fully explain the male/female discrepancy. Rather than searching for the causes of heightened risk in males, one might look, instead, for female protective factors. Girls who are diagnosed with ASD carry significantly more genetic load than boys (35), suggesting adaptive mechanisms such as the ability to effectively mask ASD symptoms (36). If such mechanisms exist, they remain to be discovered. There are also several clinical differences between boys and girls with ASD: more externalizing behavior (e.g., aggressive outbursts, social impairment, hyperactivity, restricted behaviors and interests) in boys and more internalizing symptoms in girls (e.g., depression, anxiety, emotional symptoms) (37). Since diagnostic criteria are based on the much more prevalent male presentation, it is likely that girls, who present somewhat differently, are underdiagnosed (38).

### Eating Disorders

If one omits neurodevelopmental disorders such as ASD, the male/female prevalence ratio is higher in eating disorders than in any other forms of mental illness. Anorexia nervosa affects 0.9–4% of women and roughly 0.3% of men (39). This major difference in prevalence rate is usually attributed not to biology but to contrasts in the gender roles assumed by women and men in adolescence and to the peer pressures to which they are exposed (40). Multiple interacting factors, however, probably play a part. Genetic and epigenetic causes, gonadal hormone levels, and constituents of the immune system have all been considered as potentially contributory to the prevalence ratio. In other words, socialization into gender-specific roles and societal expectations may be exaggerating innate proclivities (41). Because thinness in many cultures equates to female beauty, and beauty serves as a proxy for youth, health and reproductive fitness, eating disorders may, from an evolutionary perspective, reflect an ancient mating strategy—enhancing beauty to attract men who will ensure long term survival of offspring (42). In modern times, this strategy is made increasingly complex because media and the fashion industry define beauty standards.

### Posttraumatic Stress Disorder

In most prevalence studies, approximately twice as many women as men are diagnosed with PTSD, and this ratio is consistent across diagnostic systems, measurement methods, ethnicities, cultural backgrounds, and study populations (43). The trauma preceding this disorder is considered to be an exposure to one or more extremely threatening events, such as sexual, physical, or severe emotional abuse, a death in the family, or a serious accident, natural or manmade disaster (44). Symptoms of PTSD are more severe, chronic, and recurrent in women than they are in men (45–47). Associated symptoms/behaviors also differ. Women with PTSD in addition to the core symptoms report dissociative phenomena, somatization, disordered eating, low self-esteem, guilt, and shame. Men, in contrast, present with problems relating to aggression, thrill-seeking, risk-taking, and impulse-control (7). Diagnosable comorbidities induced by trauma also differentiate men and women -e.g., depressive and anxiety disorders accompany PTSD in women while substance use disorders are prevalent in men (48).

We will now explore in greater detail some of the theories that have been proposed to explain sex and gender differences in mental illnesses, using our three selected disorders as examples. The emphasis in each of the theories is different, but they all acknowledge that they are, in themselves, incomplete, and that complementary perspectives are needed for a full explanation of the many factors that result in sex/gender differences.

## Sex-Based Theories

### Sex Chromosomes

At conception, most females possess two X chromosomes and most males have one X and one Y. Some male-specific genes are located on the Y chromosome, the most important one being the SRY or testis-determining gene. Once testes develop, the process of subsequent differentiation between the sexes begins (49). Male testes and female ovaries secrete different levels of hormones (estrogens, androgens, progestins, and anti-Müllerian hormone) that subsequently influence most body organs, including the brain. Because females have two X chromosomes, one in each body cell is automatically and randomly inactivated so that males and females end up with an approximately equal total of gene products (50). But ~25% of genes on the inactivated X chromosome escape full inactivation. This process, in addition to epigenetic effects on genes on all chromosomes (50, 51), results in sex/gender differences in the rates of many medical conditions. Proposed mechanisms include the unequal level of gene products expressed by sex biased genes. These are genes that are present in both sexes but that express different quantities of protein products in men and women due to the impact of either epigenetic factors or sex hormone levels (52).

Genes are important even in conditions such as eating disorders, which result from mainly social causes. Twin-based heritability for anorexia nervosa ranges from 48 to 74%, for bulimia nervosa from 55 to 62%, and for binge-eating disorder from 39 to 45% (53). A recent genome-wide association study (GWAS) of ~17,000 anorexia nervosa cases and 55,000 controls yielded 8 significant genetic loci that may contribute to etiology (54). Susceptibility genes, it should be noted, may lie dormant for long periods and only become expressed under specific dietary or perhaps hormonal conditions (55). Sex chromosomes have only recently begun to be included in GWAS screens. In addition, many of the databases used for transcriptome analysis are skewed toward data that has been generated predominantly in males, which means that more genes relevant to sex differences in medical conditions still await discovery (56).

### Brain Structure

Sex biased genes are present in the central nervous system in great number and may be partially responsible for the neuroanatomical sex differences that have been reported in the human brain. Brain structure develops not only through the action of genes, but also in response to environmental influence so that postnatal experience, which usually differs significantly in girls and boys, affects the size of specialized brain regions. In spite of many reported sex/gender differences in both animal and human brains, these are subject to considerable heterogeneity, even within regions, so that the human brain is not male or female, but best described as a mosaic of relative masculinity and femininity (57). Nonetheless, the question remains as to whether early life programming by testosterone in male fetuses leads to enduring sex differences in brain and behavior (33). Girls who experience elevated androgens *in utero* tend to display prototypic male behaviors to a variable degree that does not necessarily correlate with structural changes in the genitalia (58). For instance, persons who are born completely insensitive to androgen can be chromosomally XY but phenotypically and psychosexually female (59). This is important because it means that in humans, as in experimental animals, the lack of androgens, rather than the presence of ovarian steroids, is what principally determines femininity.

The developmental trajectory of the brain differs by region and by sex such that the pace of maturation of large areas of the brain, such as the prefrontal cortex and the hippocampus, differs in boys and girls (60, 61). As a result, experiences of trauma or injury, though occurring at comparable ages, may manifest very differently in the two sexes/genders (62). In general, the pace of maturation and development of the nervous system proceeds at a faster pace in girls (63) and, at puberty, comes under the influence of estrogens, which exert protective effects on brain systems via reduction in neuro-inflammation, promotion of synaptic plasticity, and regulation of neurotransmitter signals (64). Growing scientific attention is also being given to sex-based differences in brain circuitry (65), which probably affect response to environmental insult. This means that, although differences in the brain are commonly considered the product of biological sex, gender-based influences may also play a strong, if not equal, role (66).

### The Central Nervous System and HPA-Axis

The central nervous system (CNS) and the hypothalamic-pituitary-adrenal (HPA) axis are both central to the body's response to stress. The role of the CNS is to respond quickly and efficiently to perceived stressors and the role of the HPA axis is to maintain that response for as long as needed, and to return the body to homeostasis once the threat has subsided (67, 68). The HPA axis connects the CNS to the endocrine system while also impacting the sympathetic-adrenal medullary (SAM) axis, the immune system, and the cardiovascular system, as well as directly modulating affective and cognitive processes in the brain (69, 70). A dysregulated HPA axis is central to the trauma and stress response. Dysregulation can result in either an inadequate response to threat and or an excessive stress response that persists long after the threat has passed; both are potentially harmful (71). As a result, the HPA axis is hypothesized to strongly influence the development of stress- and trauma-related disorders such as PTSD. Furthermore, a dysregulated HPA axis can help to explain why people suffering from PTSD respond to traumatic triggers either with hyper-arousal (increased heart rate, respiration, and blood pressure), often resulting in a startle response and a feeling of panic, or with hypo-arousal (decreased heart rate, immobility), a frequent precursor to dissociation. Interestingly, the former response appears more common in men and the latter in women (71), for unknown reasons.

The development and functioning of the HPA axis are influenced by one's genome, the prenatal environment, and by life experiences. HPA functioning is especially sensitive to repeated early life trauma (72). As a result, there are multiple ways in which both sex and gender can affect the HPA axis. Both adrenocorticotropic hormone (ACTH) and free cortisol levels have been reported to increase twice as much in men as in women in response to a psychosocial stress task, though results may depend on the specific stressor studied (69). Some studies have suggested that women's monthly hormonal fluctuations sensitize the HPA-axis (73, 74). This may be one reason why women, more than men, risk developing stress-and-trauma-related disorders. In ASD, on the other hand, multiple lines of evidence suggest that dysregulated steroidogenesis during early development contributes to male preponderance (33, 34). With respect to eating disorders, an activated HPA axis has been closely associated with these conditions although the activation may be secondary, an effect of starvation (75).

### Somatic Factors

Somatic factors outside the brain, such as immune processes, are capable of influencing the brain and resulting in psychiatric symptoms. In most animals, male sex is associated with a relatively lowered immune response and a relatively higher susceptibility to infection (76). The explanation may lie in the many immune-related genes that are encoded on the X chromosome. Although, as explained earlier, one of the two copies in females is silenced, immune-related genes may escape inactivation in some body cells. As well, immune cells such as T cells and B cells show varying sex-related rates of age-associated decline, with immunosenescence starting earlier in males than in females by ~6 years (76). Furthermore, female hormones play an important protective role in immunity. Estradiol, for instance, has been convincingly shown to reduce the levels of inflammatory cytokines (77). This accords with preclinical animal research, which finds both greater levels of inflammatory signaling molecules and activated immune cells in healthy males than in healthy females (78). From an evolutionary perspective, immune sex differences serve an important purpose because the female immune system needs to suppress an antibody response to male sperm during conception and to male fetuses during pregnancy (79).

Sex differences in the immune system affect the brain, and are seen most notably in the functions of astroglia and microglia, both brain cells that reportedly play a role in psychiatric disease (80). It is currently believed that a combination of genetic sex, plus the organizational and activational effects of gonadal hormones, plus levels of stress hormones contributes significantly to brain immune function (81).

Inflammation experienced either *in utero* or very early in life is a known risk factor for ASD (81–83), and transcriptome analysis of fetal human brain reveals high levels of gene expression consistent with increased baseline immune reactivity in PTSD (46, 47), which may be transmitted to offspring in a sex-specific manner through varying levels of maternal proinflammatory cytokines over the course of pregnancy (84). Animal research suggests that maternal immune activation or early life inflammation exert negative impact on the brains and subsequent behavior of males, but not females (85).

There are connections between the immune system, the brain, and the gut (86). The composition of intestinal microorganisms appears to affect brain functioning and sex/gender reportedly plays a part in that composition, although study results are inconsistent (87, 88). Men and women tend to eat different foods, take different medications, show different susceptibility to weight gain, and their colonic transit times, on average, differ. These are all factors that affect gut bacteria and potentially influence the expression of many psychiatric disorders (89–91), but this area of research is still in its infancy.

### Gonadal Hormones

Gonadal hormones play important roles in differentiating the manifestations of mental disorders in men and women (92–94). Besides conferring protection against the effects of injury, inflammation, ischemia and apoptosis, estrogens also promote neuronal growth and enhance neuro-reparative processes such as remyelination. They also serve important regulatory functions in relation to neurotransmitter systems (94). There is evidence that gonadal hormones play a critical role in aiding the epigenetic expression of eating disorder genes (95–97). Gonadal hormones also contribute to our understanding of ASD (98) and PTSD (99).

Though findings are confounded by differences in measurement methods and ascertainment times, time periods associated with female reproduction, such as pregnancy, the postpartum, and menopause are often associated with the emergence or aggravation of psychiatric symptoms (100). For example, women exposed to traumatic events during the early follicular phase of their menstrual cycle show comparatively increased risk of developing PTSD (100). Naturally occurring hormonal fluctuations in women can also affect response to treatment with psychiatric medications (101). The efficacy of certain psychotropic medications appears to decrease during the luteal phase of the menstrual cycle (102) and the risk of drop-outs from trauma-focused cognitive behavior therapy varies according to the time of the month in which it is initiated (7).

An important limitation to note when discussing the influences of gonadal hormones on symptom development is that much of the data is based on animal studies and requires further study in humans (101).

## Gender-Based Theories

### Parental Expectations

Whereas, sex differentiation is initiated at conception, the influences of gender start to take effect after birth. Parental expectations of offspring, however, precede birth. Parents choose nursery decor, infant clothing, distinctive toys and given names to match cultural gender norms. For example, in many cultures, girls' names allude to beauty and grace while boys' names suggest strength and sturdiness (103). Differentiation along similar lines is widely accepted, as shown by results of a recent study from China. College students, when asked to rate forenames, characterized feminine names as reflecting warmth and masculine names as representing competence (104).

Gender norms are defined as the expectation that boys and girls, men and women, think, feel, talk, and generally behave in gendered ways. The content and rigidity of gender norms and their endorsement by parents and peers vary across cultures and sub-cultures, but the general rule is that boys are expected to be strong and to control their emotions (except anger, which is permitted) whereas girls throughout the world are expected to be amiable, relatively docile, and nurturing (8). Gender traits are often grouped together and referred to as femininity vs. masculinity.

### Gender Socialization

Children learn to act and to think according to gender norms, with parents considered to be the primary channel through which these norms are transmitted (105). Parents interact differently with girl and boy children, encouraging more physical exploration and toughness in boys and more emotional communication, tenderness, and focus on appearance in girls (106). This has been seen as contributing to the high prevalence of eating disorders in girls and women. Parental expectations are reinforced by interpersonal interactions and by the media and can lead to the development, in girls, of vulnerability to body image concerns. Though this is a generality, gender norms frequently encourage girls and women to aim for an idealized body shape and boys and men to nourish a drive for muscularity, accomplishment and power (107, 108).

In general, children are rewarded for what the world around them judges to be desirable behavior and criticized for behaviors that are idiosyncratic (109). In terms of gender socialization, this means that society, including one's parents, teachers, and peers, have low tolerance for personality traits and characteristics traditionally assigned to the opposite gender. Over time, such socialization processes strongly impact the way children see themselves. This happens through mechanisms such as the Rosenthal (Pygmalion) effect (110). Phenomena such as the frequently impaired communicative abilities in boys and underdeveloped mathematical abilities in girls have been linked to this effect.

Stress arises when the environment poses threats to the gender norms that one's culture values. For this reason, men and women are to an extent stressed by different kinds of threats. Women, reportedly, are most threatened by relationship losses whereas threats to status and financial stability are especially stressful for men (111).

### Gender Roles

Children internalize the gender norms to which they are exposed and, over time, impose these stereotypes on themselves, a phenomenon referred to as self-socialization (112). Children especially take on gender roles modeled by same-sex adults and peers whom they admire and this affects their actions, appearance, hobbies, dreams, educational pursuits, careers, intimate relationships, and eventual parenting styles (113). Structural theories suggest that different gender roles lead to exposure to different kinds of threats and stressors, each calling for different coping strategies, and resulting in gender differences in adaptability and mental health (114). One such theory, role restraint theory, states that, when men and women are allowed to take on similar roles and face similar stressors, differences in their responses disappear (115). Socialization theories address not only exposures but also the development of sex-specific vulnerabilities to stressors and sex-specific coping strategies, e.g., women tending to confide and share, men tending to deny and seek distraction (116).

### Gender Identity

Though the term “gender identity” is sometimes used to refer to self-perceived gender, it is best defined as a person's deeply felt internal and personal experience of their own gender (117). This includes how one sees oneself, the possibilities and restraints attributable to one's gender, a sense of one's body, societal role, and sexual preferences. It also includes non-binary gender identification (118). Gender identity is most lastingly developed during adolescence, with body image, sexual orientation, and range of potential gender roles becoming central themes (119). A perceived incongruence between society's gender norms and one's gender identity can become a source of emotional problems and felt constraints. For example, masculine gender-role stress, defined as stress resulting from the struggle to live up to masculine ideals, has been associated with PTSD (1, 120). A striking example would be a male veteran who suffers from PTSD after a sexual assault and struggles with the perceived discrepancy between military masculine ideals and the role of victimhood and someone struggling with mental illness (120).

### Diagnostic Bias

There are multiple sources of gender bias both in mental health research and in the clinical setting. Women have traditionally not been widely recruited for biologically-based mental health research studies because their hormonal fluctuations complicate the task of appropriate matching and because of the concern that an experimental intervention may potentially harm the fetus should an unexpected pregnancy occur. This means that clinical guidance is often still based on research involving mainly men (7). In contrast, many psychotherapeutic trials are conducted primarily on women, simply because more women than men seek help for mental health problems. Furthermore, the vast majority of PTSD research on men is conducted on military personnel or veterans and may not be generalizable to civilian men, let alone to women.

Even in a biologically based disorder, such as ASD, there is evidence of diagnostic bias. This is likely due to the fact that the earliest cases of these disorders were observed in boys, so that the diagnostic criteria were established based on characteristic male symptoms. As stated earlier, it is perhaps because of this that ASD remains often undetected in girls, especially when symptoms are mild (37, 121). Sources of data have substantial clinical impact on how diagnostic criteria are formulated, on screening tools, on diagnostic and clinical guidelines, and on the efficacy of the many different ways in which mental illnesses are treated. Most standardized tests, diagnostic questionnaires, and diagnostic criteria themselves are based on the assumption that men and women experience symptoms in the same way and that a specific question is equally well-suited for eliciting relevant symptoms in women and men, whereas this may not be the case (122). In contrast, in the absence of standardized measures, clinicians may preferentially ask questions of one sex/gender and not of the other. For example, women (but not men) are commonly asked during assessments about exposure to sexual trauma and perinatal loss, though men, too, are exposed to such experiences and their sequelae. The same is true for questions about spousal abuse. Women, on the other hand, are unlikely to be asked about ever engaging in acts of aggression. Similar to the Rosenthal effect mentioned previously, when men and women are repeatedly asked questions about specific experiences and/or symptoms, in time, they will become more likely to report such symptoms whereas, when these areas are left unprobed, they stay unreported, especially so if they are perceived as incongruent with gender norms (115). Thus, clinicians may be more likely to overlook the presence of certain disorders in one sex compared to the other.

Furthermore, using dichotomous categories to assess sex and gender forces both male and female study participants into highly heterogenous groups, with no allowance for intra-group variability. It may be time to rethink how we assess sex and gender and introduce continuous measures, allowing for a wider range of gender-normative variability.

## The Coming Together of Sex and Gender

As can be gathered from the literature we have cited, it is essentially impossible to state with certainty which differences between male/female expression of illness are primarily based in biology and which are products of nurture, learning, or environment. For most mental health syndromes, there is evidence for the impact of both sex and gender, through the different factors summarized in Tables 2, 3. Some distinctions between the two influences may, however, be hypothesized. The fact, for instance, that sex/gender differences are found across cultures favors sex-related explanations, whereas cross-cultural variations in the prevalence of differences suggest the influence of gender. In most cases, including the three disorders we focus on here, though sex/gender differences are found universally, their strength varies somewhat across regional and cultural groups, indicating that both sex (innate vulnerability) and gender (specific exposures and expectations) play a part (123, 124). Because most published research has been conducted in Western cultures, relatively little is known of the extent to which results from studies on gender-based factors, such as gender identity and gender-role stress, generalize across cultures. For example, whereas the negative effects of masculine gender-role stress are relatively well-established within Western culture, it is possible that feminine gender-role stress may be clinically significant in parts of the world where women's roles are more restricted. Indeed, many culture-bound syndromes (125–127) can be considered as expressions of women's difficulties with their assigned gender roles.

**Table 2:** Summary of sex-based theories.

• Responsible genes may present only on the X or Y chromosome or be expressed differently due to escape from X inactivation or epigenetic influences
• Sex differences may exist in brain anatomy, circuitry, function, or output, causing the brain to be viewed, not as dichotomous, but as a mosaic of relative maleness and femaleness
• Functional sex differences exist in the CNS, HPA-axis, SAM-axis
• Sex differences in the immune system, cardiovascular system, and gut exert influence on brain pathways
• Gonadal hormones influence the human experience throughout life, especially through puberty, the menstrual cycle, pregnancy, the postpartum period, lactation, and menopause and through use of hormone-based contraceptives, hormone-replacement therapy at menopause, and fertility hormones in women, and through hormones used for gender affirming care and treatment of transgender individuals
• Adrenal androgens and estrogen both exert protective effects against affective and anxiety disorders
• Both endogenous and exogenous gonadal hormones may influence treatment efficacy in women
• Hormonal instability may in itself pose a risk to the mental health of women by destabilizing homeostasis

**Table 3:** Summary of gender-based theories.

• Infants are often born into a world that has already been prepared for them in accordance with their sex
• Children grow up in the context of gender, as they are met with gender-based expectations, rules, norms, bias, and interpersonal behavior
• Children internalize cultural gender norms in terms of gender-based schemas, gender roles, and gender identity, which affect actions, hobbies, dreams, choice of education and career, intimate relationship behavior, etc.
• Whereas, femininity does not appear to influence mental health, low levels of masculinity, gender-role stress, and gender minority status are associated with mental disorders
• There are multiple sources of gender-based bias in both clinical practice and research that limit our ability to draw conclusions on the influences of sex and gender, generalize research findings, and properly treat mental disorders
• A first step toward illuminating the importance of sex and gender in research may be to properly assess the two in the first place and to begin measuring sex/gender in non-dichotomous ways

In most cases, when looking for etiological strands leading to mental illnesses, it is impossible to disentangle the many interacting influences of sex vs. gender. For example. though sex/gender differences in “masculine” and “feminine” traits are rooted in biological sex, socialization processes prod girls to adopt traits assigned to femininity and boys to model traits generally assigned to masculinity (112). Furthermore, the sudden increase of sex/gender differences around the time of puberty in many mental disorders is often interpreted as support for the influence of gonadal hormones, but to what degree hormones directly contribute to this finding is unclear. Of probably equal importance is the increased exposure in adolescence to societal pressures to conform to gender-role expectations (72). Evidence for the impact of both sex and gender is present in almost all such attributions.

It is also important to note that sex- and gender-based influences often modulate each other. For example, prenatal gonadal hormones affect gender identity and sexuality, which, along with secondary sex characteristics and hormones, lead to different socialization experiences, which, in their turn, impact the body's gonadal hormone production (128, 129). As such, sex and gender appear to influence each other in multiple bidirectional ways. In general, both brain functions and HPA-axis reactivity are affected by genetic predisposition, biological development, and environmental exposures/expectations (72).

In the following section, we build on the observations presented above to suggest how sex and gender based influences coalesce.

## Discussion

We have cited literature demonstrating multiple potential explanations for men's and women's different vulnerabilities to specific psychiatric disorders. On the basis of such explanations, several theoretical models have been constructed, of which mediation models form one subgroup. Mediation models are built on the premise of risk factors and protective factors. For example, in one such model (130), most of the variance of sex/gender in PTSD was attributed to pre-existing symptoms and traits, peri-traumatic reactions, and subsequent negative thoughts, on one hand, and absence of social support on the other (130). Such models, however, generally fail to explain why, as a group, men and women differ in the all-important risk and protective factors. We, thus, run the risk of tautological arguments (e.g., women have higher symptom levels after a traumatic event because they had higher symptom levels prior to the event). One way of arriving at more accurate models is to analyze the roots of sex- and gender-related mediators, be they risk or good fortune. For example, whereas the presence of substantial social support may distinguish women from men, we still need to understand why men and women elicit different levels of social support, use their supports for different purposes, and appraise their helpfulness in different ways. As stated by Kistner, naming mediators is not sufficient (11). Instead, we need to better understand how sex/gender differences in both distal and proximal risk factors arise.

In what follows, we examine how exactly sex and gender come to be associated with specific sex differences in mental disorders by proposing a model that combines multiple sex and gender based influences on mental health.

### A Diathesis-Stress Model for Understanding Sex and Gender Differences in Mental Disorders

Diathesis-stress models have been used across disciplines to integrate multiple baseline vulnerabilities or predispositions, so-called diatheses, with situational stressors (triggers) (131). According to these models, whether or not a specific person develops a disorder depends upon the interaction between pre-existing vulnerabilities and the extent, number, timing, and severity of the stressors they experience.

**Figure 1:**
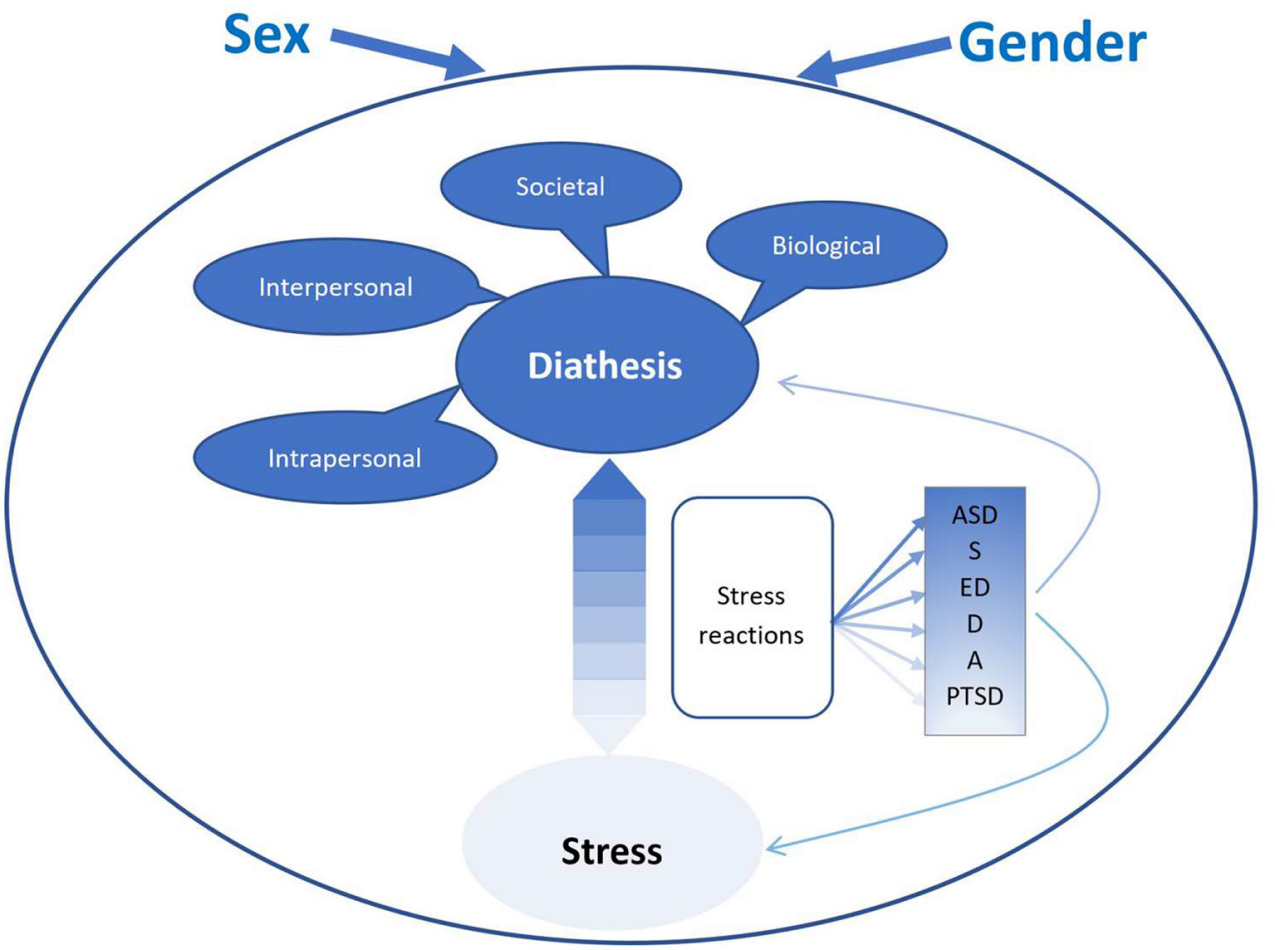
A diathesis stress model of sex and gender differences in mental disorders. ASD, autism spectrum disorder; S, schizophrenia; ED, eating disorders; D, depression; A, anxiety; PTSD, posttraumatic stress disorder. The differences in shading represents a shift in the diathesis vs. stress balance for the different disoders.

We propose a diathesis-stress model for sex and gender differences across mental disorders (though we illustrate the model via our three selected disorders—ASD, eating disorders, and PTSD). As indicated in Figure 1 through the use of color gradations, the emphasis on diathesis vs stress varies according to disorder. A diagnostic category thought to originate mostly from diatheses is shown in a darker blue than one mostly caused by exposure to stressors and traumatic events. For example, ASD shows substantial heritability and results mainly from biological causes (132). Moreover, it emerges very early in life before many environmental stresses have had a chance to accumulate. However, *in utero* stressors, are also part of the genesis of ASD (133), thus a diathesis-stress model is appropriate. Furthermore, disruptions in everyday life and comorbidities elicit symptoms and, thus, are central to the expression and quality of life in ASD (134). In contrast, PTSD requires exposure to a traumatic event and cannot, by definition, develop in the absence of such an event. However, whereas highly resilient individuals may only develop PTSD after a particularly toxic traumatic event, extensive underlying diatheses can cause others to succumb in response to relatively minor stresses. Finally, though most investigators consider social pressures to be the main factors causing eating disorders, there is evidence for substantial individual predispositions (135), thus placing eating disorders in between ASD and PTSD. Please see Table 4.

**Table 4:** Examples of how the diathesis-stress model may account for sex/gender differences in ASD, eating disorders, and PTSD.

**Autism spectrum disorders**
Biological diatheses
• ASD has a strong biological basis with sex differences being attributed to dysregulated steroidogenesis during early development, testosterone levels, and brain differences (i.e., “Extreme Male Brain Theory”).
• ASD may require more genetic load in girls than boys
Intrapersonal diatheses
• Higher levels of aggression in boys may lead to more aggressive outbursts
• Girls/women may be more adept at masking, and possibly compensating for, ASD symptoms
Interpersonal diatheses
• More externalizing behavior in boys may increase social exclusion and bullying, increasing alienation and social withdrawal and reducing quality of life. More prosocial behavior in girls may reduce this.
Societal diatheses
• Diagnostic bias may cause ASD to be overlooked in girls/women—and possibly over-diagnosed in boys/men
Stressors
• Pre-natal or early postnatal inflammation in boys may serve as a stressor that may, in some cases, serve as a triggering stressor
• Smaller everyday life stressors may affect functioning and quality of life, though the impact on sex/gender differences in ASD is unknown.
Peri-/*post-*stressor factors
• Boys may be more likely to become outwardly aggressive in response to stressors
**Eating disorders**
Biological diatheses
• Though biological diatheses are believed to be less involved than in ASD and PTSD, genetic factors, female gonadal hormones, the immune system, and intestinal bacteria may help increase the risk for eating disorders in girls
• Epigenetic processes may be triggered by dieting behavior and the onset of puberty in girls and women
Intrapersonal diatheses
• Internalized beauty standards in young girls and women call for more strive for thinness and dieting behavior
• Negative affectivity and rumination, both more common in girls, are risk factors for eating disorders
Interpersonal diatheses
• Peer pressure and other group dynamics are believed to contribute more to problematic dieting behavior in girls than boys
Societal diatheses
• Gender roles and societal expectations, including beauty standards in many cultures, of young women are believed to be the largest contributor to eating disorders in girls and women
• Cultural changes in eating habits with increased intake in high caloric and sugary foods may contribute to increases in some cultures, though the specific influence on sex/gender differences is unknown
Stressors
• External pressures and stressors (e.g., early mealtime conflicts, bullying, body criticism) are believed to contribute substantially to the genesis of eating disorders. In most cases, it is believed that it is the accumulated effects of such stressors, that contribute to the development of eating disorders. Thus, most such stressors will contribute to symptom development as diatheses.
• External stressors may trigger specific episodes of binge-eating, purging, or fasting
• Exposure to traumatic events have been found to increase the risk for eating disorders. This may especially be the case for events associated with high levels of shame and disgust, such as sexual assault/abuse
Peri-/*post-*stressor factors
• Problematic eating behavior and dieting as a way of coping with stress are more common in girls and are often precursors to eating disorders
**PTSD**
Biological diatheses
• Women are believed to have an inherent preparedness to respond to stress with excessive fear and anxiety, making them more vulnerable to trauma
• The HPA-axis is believed to become more easily dysregulated in women
• Female gonadal hormones, and perhaps especially the constant fluctuations in these, are believed to contribute to their higher risk of developing PTSD
Intrapersonal diatheses
• Multiple intrapersonal risk factors (e.g., negative affectivity, rumination, low self-esteem, depression) are more common in girls
Interpersonal diatheses
• Though women receive more social support, they are also exposed to more negative social responses (e.g., victim blaming) than men
• Women are indirectly exposed to more traumatic events than men, as others share their trauma with them
Societal diatheses
• Boys are encouraged to confront their fears more than girls, which has been speculated to reduce avoidance behavior in boys but induce learned helplessness in girls
• PTSD may be under-diagnosed in men, likely partly due to men not seeking help and under-reporting symptoms, both of which may be related to gender norms (and to masculine gender-role stress)
• Certain potentially traumatic events (e.g., sexual assault/abuse, infant loss) are believed to be under-reported in men, causing PTSD to go undetected for longer
Stressors
• Women are exposed to more potentially traumatic events associated with high risk of PTSD, such as rape, childhood sexual abuse, and betrayal trauma
Peri-/*post-*stressor factors
• Women are more likely to than men to respond to potentially traumatic events with intense fear, horror, helplessness, dissociation, and negative appraisals, all of which are risk factors for PTSD
• Trauma-related guilt and shame are more common in women and known risk factors for PTSD

Few models have addressed the potential effects of multiple diatheses in combination with an accumulation of situational stressors. Such a model (131), however, is truer to life given that the etiology of psychiatric disorders is multifactorial and complex. One complexity is the usual categorization of sex and gender either as a diathesis or as a risk factor (136), but this is an oversimplification because sex/gender exerts its combined effects through a relatively large number of mediating factors (136).

Hyde and colleagues (137) constructed an integrated model of the emergence of sex/gender differences in depression in which they hypothesize that **a**ffective, **b**iological, and **c**ognitive factors converge to result in a vulnerability that is only expressed when a severe stressor triggers symptoms, the so-called ABC model (137). Factors **a**, **b**, and **c** interact with each other and with sex/gender to form the diathesis. The presence of a particularly strong risk factor within one of these domains may be sufficient for symptoms to emerge. Equally possible is for relatively weak risk factors across two or three domains to combine and, together, trigger depression. Although Hyde et al.'s model focuses on depression, a similar model can be constructed for other disorders, including those that first emerge in childhood (e.g., ASD) and those that most commonly emerge in adolescence (eating disorders) or adulthood (PTSD).

We suggest that a diathesis-stress model can be created to account for the complex interaction between sex and gender and their combined involvement in the development and expression of multiple mental disorders. Whereas, some aspects of the model will be specific to certain disorders, many elements will be relevant across diagnoses. We will now introduce some of the key elements of such a model.

### Diatheses

In accordance with a model presented by Parker and Brotchie (138), designed to account for sex/gender differences in depression, Christiansen (1) first suggested that the diathesis component of the model represents an inherent preparedness toward responding to a stressor with excessive fear and anxiety, which, when a person is exposed to trauma, significantly increases the risk of developing symptoms (1, 138). This inherent preparedness is, on average, more marked in women than in men and, thus, represents a biological sex difference in PTSD vulnerability (139). There is evidence that women, as a group, have a greater likelihood than men to be genetically predisposed toward fear (140). In line with other models (130, 131), we acknowledge that diatheses may vary in their degree of specificity, with some being disorder-specific (e.g., early childhood mealtime conflicts as a specific risk factor for eating disorder in adolescence), while others are shared among multiple disorders (e.g., negative affectivity or childhood trauma) (141, 142). Elwood and colleagues (136) suggest a possible hierarchy of diatheses that are more or less essential for the development of a specific disorder. Their model supports our earlier statement, that a single high-level diathesis may be sufficient for a person to develop a disorder when exposed to stress, whereas a lower-level (less specific) diathesis can only lead to symptoms when combined with other pre-existing vulnerabilities.

McKeever and Huff (131), in their diathesis-stress model of PTSD, suggest two types of diatheses: ecological and biological, which influence each other. Moreover, either one can elicit specific stressors from the environment. Ecological diatheses consist of risk factors linked to an individual's self (e.g., developmental history, coping mechanisms, social support). The distinction between ecological and biological diatheses is particularly relevant in a model of sex/gender differences, as gender would probably be considered an ecological diathesis and sex a biological diathesis, depending on the disorder. The distinction is further relevant to a model spanning multiple disorders, with some being biologically influenced (e.g., ASD) and others (e.g., PTSD) being more ecologically based. Our model distinguishes among different ecological diatheses by separating out intrapersonal factors, interpersonal/relational factors, and societal factors. Our model also differentiates between sex and gender because we believe that biological diatheses will mostly (but not exclusively) rely on sex, whereas societal diatheses will mostly rely on gender, and intra- and interpersonal factors will likely rely equally on both. A non-conclusive list of diatheses related to sex/gender differences in mental disorders can be found in Table 5.

**Table 5:** Examples of diatheses relevant to sex differences.

**Biological:**
• Chromosomal, genetic, and epigenetic influences, including family genetic predispositions to certain disorders
• Brain anatomy, circuitry, function, or output, especially relating to the amygdala, hippocampus, or pre-frontal cortex
• Dysregulation of important systems/organs, including the CNS, HPA axis, SAM axis, immune system, gut, metabolism
• High/low levels of certain neurotransmitters, peptides, hormones, including gonadal hormones
• Exogenous influences such as food, substances, hormone-based contraceptives
• Biological and physical consequences of prior stressful and traumatic exposure (e.g., physical trauma, pain, dysregulated HPA axis)
• Overall health, including pre-existing injuries, diseases, and health complications
Intrapersonal:
• Affective factors, including personality style and temperament, negative affectivity/neuroticism, introversion, anxiety sensitivity
• Cognitive factors, including cognitive bias, schemas, appraisal, coping style, rumination
• Factors related to self and identity, including self-worth, self-confidence, bodily self-image
• Psychological defenses, such as dissociation, somatization, splitting
• Gender-related concepts, such as internalized gender roles, masculinity, femininity, gender identity, sexual preferences
• Intrapersonal consequences of prior stressful and traumatic exposure (e.g., self-blame, doubt, survivor's guilt)
• Pre-existing symptoms and disorders
Interpersonal:
• Attachment (e.g., attachment anxiety and avoidance)
• Social relations, including social networks, positive and negative social support
• Presence and quality of close interpersonal relations (romantic partners, family, friendships)
• Repetitive or prior negative social interactions (e.g., bullying, domestic abuse, ostracism, loneliness)
• Boundaries, forgiveness, diplomacy, social intelligence
• Affiliative, supportive, helpful, and help-seeking behavior
• Interpersonal consequences of prior stressful and traumatic exposures (e.g., diminished trust, isolation)
Societal:
• Minority status, socio-economic status, living situation, educational level, occupational status
• Degree of independence, marginalization,
• Access to financial resources, support, protection, medical and psychological treatment, police assistance, justice
• Societal attitudes toward exposure to distribution of work, minor stressors, violence
• Type and rigidity of gender norms and identities including wide-spread prejudice (e.g., homophobia, transphobia, misogyny, misandry) and related concepts (e.g., systemic racism)
• Prior stressful and traumatic environmental exposures, including upbringing, living in violence-prone areas, pollution, and poisonous agents

Finally, whereas diathesis-stress models generally focus specifically on personal vulnerabilities to disorders, we think of the presence or absence of external resources also being diatheses. An example would be the presence of social capital serving as protective factor against symptom development (143).

### Biological Diatheses

Biological vulnerabilities in any one disorder are often specific to that disorder, but some are more general. One example is the involvement of the same neurotransmitter in multiple disorders, which is why selective serotonin re-uptake inhibitors (SSRIs), for instance, are effective in several different categories of mental disorder. Biological vulnerabilities relevant to sex/gender differences are likely to include genes on X and Y chromosomes (but also on autosomes), impairments of brain structure and function, insufficient or excessive levels of gonadal and other hormones, disturbances of HPA axis reactivity, immune system, and/or microbiome irregularities, as addressed earlier. Biological diatheses are often present at birth and strongly impacted by sex. However, gender also affects biological factors through experience, particularly through the influence of socialization and gender-specific stressors that lead, for instance, to the activation of the HPA axis or to epigenetic arousal of dormant genes.

### Intrapersonal Diatheses

Intrapersonal diatheses include affective and cognitive vulnerabilities that, along with pre-existing symptoms and disorders, serve as predispositions to new disorders. Affective factors consist of temperament, negative affectivity, sometimes referred to as neuroticism, and mood. Most research agrees that women score higher than men on measures such as sadness, grief, negative affectivity, and anxiety proneness, whereas men tend to score higher on measures of aggression, anger, and impulsivity. Common cognitive vulnerabilities include rumination, maladaptive coping styles, poor self-perceived coping efficacy, negative appraisals, and negative schemas. Cognitive schemas are best understood as internal working models of how individuals view themselves, other people, and the world. Understanding gender norms, gender roles and gender-related schemas, and one's expectations of oneself and others fall into this category.

In our model, coping strategies and appraisals are considered post-stressor factors in that they are often situationally specific and associated with a particular stressor and a person's response to that stressor, as well as with subsequent symptoms. However, there are overall general coping styles and attributional styles that remain relatively stable over a person's life, and, as such, should be considered diatheses. For instance, women have repeatedly been found to ruminate more than men, to appraise situations as more threatening/severe than men do, and to score higher on maladaptive aspects of emotion-focused coping (144). These are enduring traits that serve as diatheses for all internalizing disorders. Furthermore, they exacerbate existing disorders, including neurodevelopmental disorders such as ASD, by undermining levels of functioning and quality of life, making it more difficult for already vulnerable individuals to cope with their symptoms. By contrast, enduring tendencies toward anger, aggression, and lack of impulse control serve as diatheses for externalizing disorders. Finally, our model also places internalized gender roles, masculine and feminine traits, gender identification, and sexual preferences into the category of intrapersonal vulnerabilities.

### Interpersonal Diatheses

Interpersonal aspects of our model include sex/gender differences related to insecure attachment, poor or negative social support (145), and victim blaming (146). Attachment is usually considered an intrapersonal factor, but we have included it in this category, as the quality of early attachments exerts a strong influence on subsequent relationships. Though women, as a group, are not less securely attached than men, they do score higher on the attachment anxiety dimension of attachment, which has been linked to later symptom development (147). With respect to social support, women have consistently been found to be recipients of both positive and negative social support, more so than men. Though positive support serves to buffer the impact of stressors, the effects of negative support (hurtful social interactions) may carry greater weight and undermine mental health.

Recurrent patterns of intimate interpersonal interactions with friends, family members, and lovers (e.g., abusive relationships, response to sexual intimacy, enjoyment of sex, relationship gender roles) fall into the category of interpersonal diatheses, whereas we categorize one off stressful interactions (e.g., fights, assaults) as stressors.

### Societal Diatheses

The last group of diatheses relating to sex/gender differences in mental disorders are social and environmental: socio-economic status, caste, residential area, educational level, work status, degree of independence, marginalization, politics and other societal factors that set individuals apart. Social hierarchies affect men's and women's access to financial resources, support, protection, financial aid, medical and psychological treatment, police assistance, and legal justice and matter greatly in the aftermath of a traumatic exposure. They are strongly influenced by gender. Factors such as financial dependence, poor access to healthcare, and exposure to domestic abuse put women at a much higher risk than men for developing trauma-related disorders, even though men, as a group, are more exposed to industrial accidents, criminal victimization, and the trauma of war (7).

Gender-based socialization, gendered violence, misogyny (and, to a much lesser extent, misandry), homophobia, and transphobia, also belong in the social diathesis category, whereas the internalized version and enactment of such phenomena fit best under intrapersonal diatheses. Though we designated gender roles as intrapersonal (because they arguably result from internalized gender norms), we have called gender-role stress a social diathesis as it results from a mismatch between internalized gender identity and society's gender-based expectations.

We note in our model that sex and gender has the power to modulate societal diatheses, for example through the ability of secondary sexual characteristics, dress, hair styles, the application of cosmetics or scents, and plastic surgery to elicit and change the behavior of others, leading to changes in one's social status. Traits associated with masculinity, such as assertiveness and aggressiveness, are also able to alter established social hierarchies.

### Stressors

The stress component of our model is composed of a range of events, from severe to minor. A non-exclusive list of such events shown previously to contribute to sex/gender differences in mental disorders is shown in Table 6. Aversive childhood events such as childhood sexual abuse are associated with multiple mental disorders later in life. But even a relatively minor break in routine upsets a child with ASD and can trigger a cascade of symptoms. As proposed elsewhere (1), the stress component of this model is much more likely to be affected by gender than by sex.

**Table 6:** Examples of stressors relevant to sex differences.

**Traumatic stressors**
• Sexual abuse and assault (women)
• Betrayal trauma (women)
• Combat, war-related traumatic events, imprisonment, non-sexual violence, severe accidents, injury, life-threatening illness, witnessing severe injury/death of
others (men)
Daily or minor stressors
• Childcare, elder-care, domestic chores, family responsibilities, support provider, caregiver (women)
• Financial responsibilities, physically demanding chores, role of protector (men)
• Loss of access to children associated with partnership break-down (men)
• Microaggressions and interpersonal conflict (women)
• Financial stressors and status loss (men)

Men, on average, experience a higher number of traumatic stressors than women. These is particularly the case for non-sexual torture, physical violence, severe accidents, gang violence, and witnessing severe injuries and death of others during both war and peacetime (46, 48, 144). Women, on the other hand, experience obstetrical trauma, sexual violence, and betrayal trauma, including intimate partner violence. With respect to everyday stressors, female gender role norms result in women being more embedded than men in the social networks of family and friends. As a result, women are exposed to more non-romantic interpersonal conflicts, losses, and indirect stressors [i.e., negative events that happen to others, such as the divorce or illness and death of close friends (148)]. Though slightly dated, one study of married heterosexual American couples found that wives reported more everyday stressors than husbands, especially in terms of household stressors, family demands, demands from others, and arguments with children (148). In turn, husbands reported more work stressors, stressors related to transportation, and arguments with non-family others. These differences may be accurate but may also result from reporting bias, as men and women differ, both in term of what stresses them, and how willingly they report such stressors. According to a classical paper, unmarried women live longer than married women, whereas married men live longer than single men (149). This may no longer apply because the division of labor between wife and husband has changed markedly over the years (at least in the West), but married women still take on additional caretaker tasks in families, as well as being more affected than men by partnership and family conflict, whereas men mostly benefit from the positive social support they receive through marriage (150).

Finally, sex/gender differences in the types of traumatic and non-traumatic events that men and women are exposed to may influence the type of symptoms they develop. For example, perhaps as a result of being more commonly exposed to betrayal trauma, women may be more likely develop self-doubts that lowers their self-esteem (151). Another example referred to earlier: whereas sexual assault often causes multiple problems with intimacy, sexuality, and self-worth in both men and women, male victims may, in addition, subsequently struggle with what they perceive to be a threat to their masculinity (120).

In accordance with other diathesis-stress models (e.g., 120), only immediate stressors are represented in the stressor component of our model. Once the stress has either subsided or become chronic, it may become part of the diathesis going forward, influencing the response to subsequent stressors. For example, a specific incident of racism, sexism, or homophobia may initially serve as a stressor and subsequently contribute to societal (e.g., perceived systemic racism or institutional sexism), intrapersonal (e.g., rumination), interpersonal (e.g., problems with intimacy), and biological diatheses (e.g., a more reactive HPA axis). The reverse is also true. Diatheses contribute not only to symptomatology but also to the types of stressors to which men and women are differentially exposed. Furthermore, an acute episode of severe mental illness may itself serve as a stressor, thus perpetuating the cycle of stress and distress. In this way, the diathesis and the stressor components of our model are impacted by each another.

### Peri- and Post-stressors

Though peri- and post-stressors do not fit neatly into either the diathesis or the stressor part of the model, they play a very important role in the development of many psychiatric disorders. In the case of PTSD, two meta-analyses have found that pre-traumatic risk factors account for only a small percentage of variance in PTSD development compared to peritraumatic and posttraumatic risk factors (152). Thus, a full diathesis-stress model can fully explain sex/gender differences in PTSD only to the extent that it can find a way to include effects of risk factors occurring at the time of and following a trauma. Similarly, for other disorders, the manner in which an individual appraises, responds to, and copes with a given stressor is often central to severity and chronicity. Although very relevant to chronicity, few diathesis-stress models have explicitly included peri and post-stressor factors (153).

It could be argued that peri-stressor factors should be part of the stress component of the model, as it is not the event in itself, but rather the full experience of it, e.g., perception, appraisal, and response, that is central to symptom development. However, this solution does not accommodate post-stressor factors that are vital to the development, and especially the maintenance, of symptoms.

It is important to note that peri-and post-stressor risk factors are strongly linked to pre-stressor risk factors. How a person responds to an event is, in large part, dependent on personality, affectivity, neurobiology, and past experience. Thus, we argue here that, since peri- and post-stressor factors are simultaneously influenced by diatheses and by the type and severity of the stressor, while also contributing to the overall experience and effect of the stressor, they deserve separate representation in the model. We have linked them to the diathesis-stressor interaction by referring to them as “stress reactions,” which includes both acute and long-term responses. Acute responses are appraisals of the stressor plus the biological and psychological responses that occur during and shortly after stress exposure. More long-term responses are continued appraisal, situational coping, social support, and the reactions of others. While these stress reactions contribute to both the type and the severity of symptoms, they are also, in turn, affected by the symptoms that emerge. For example, an individual may appraise early symptom development as either a natural reaction to a traumatic event or, at the opposite extreme, as a sign of losing one's mind. This may, in turn, either decrease or increase the number and severity of symptoms, as well as affect re-appraisal of the traumatic event. Though this interaction is bidirectional during the early aftermath of the stressor, we have chosen in our illustration of the model to include only arrows going from stress reactions to disorders/symptoms in Figure 1, in order to acknowledge that, over time, this association goes mostly in one direction.

Stress reactions are particularly relevant to consider in a diathesis-stress model of sex/gender differences as men and women tend to differ widely in both their acute and their long-term reactions to stress. As previously discussed, sex/gender differences have been demonstrated in the immediate physiological and psychological stress response, e.g., in hypo- vs. hyperarousal, perception of social support, coping ability, and treatment-seeking. It is our belief that sex/gender differences in these early responses are central to the emergence of clinically relevant symptoms and to their maintenance.

## Conclusions

Figure 1 is our diathesis stress model that focuses on the role of sex and gender in psychiatric disorders. We argue that sex/gender disparities in (a) biological, intrapersonal, interpersonal, and societal diatheses, (b) everyday stressors, major life events, and traumatic events, and (c) the physiological and psychological response to such stressors, when combined together, result in sex/gender differences in the epidemiology, clinical expression, and treatment response of most, if not all, mental disorders. Thus, though the model is exemplified here with ASD, eating disorders, and PTSD, it is our belief that it may equally be used with other disorders, including, but not limited to, schizophrenia, anxiety disorders, and depression, as shown in Figure 1. It is a major strength of this model that, in addition to highlighting differences between men and women, it also takes account of the many intra-sex variations (hormone levels, sexual identity, minority status) that are sometimes used in attempts to dismiss the influence of sex/gender.

The distinctions between different groups of diatheses is admittedly somewhat artificial because they are almost always intertwined. The concept of specificity within our model is compatible with the idea of multiple pathways, consisting of different combinations of diatheses and stressors, resulting in different categories of disorders, different symptom constellations, and different comorbidities (152). As an example, the high female/male prevalence ratio of trauma-related disorders, such as PTSD anxiety disorders, depression, somatization, and dissociative disorders, may to a large extent be accounted for by shared diatheses from each of the four domains: a more reactive HPA-axis, more negative affectivity, greater exposure to negative social support, and socialization processes that reinforce learned helplessness. It is important to emphasize that learned helplessness is, indeed, *learned* and not a function of biological sex. Preclinical research shows that female rodents are *less* likely than their male counterparts to show learned helplessness (154). Women may be vulnerable because of the types of traumatic events (e.g., rape) to which they are exposed (155). In contrast, men, for reasons not yet understood, are particularly vulnerable to *in utero* and early life adversity affecting brain maturation (156).

The combination of the relative specificity of diatheses and the complex interplay of sex and gender in our model may help to explain why certain disorders tend to aggregate together (internalizing vs. externalizing disorders) and to become, to some extent, sexually dimorphic in prevalence. We believe that this adds to the clinical relevance of our model.

Specificity in terms of high- vs. low-level diatheses for a disorder opens the possibility for the establishment of a hierarchy of diatheses, which may, in the future, help with the creation of reliable vulnerability models. This would serve the cause of prevention in persons at high risk of developing specific disorders. For example, such research could enable clinicians to target interventions at high-risk disaster survivors or military veterans to help prevent the development of PTSD.

Finally, it should be noted that the diathesis-stress model may itself be moderated by sex. The tendency for women to suffer more from trauma- and stress-related disorders and for men to suffer more from neurodevelopmental disorders suggests that the overall weight of stress on one hand and diathesis on the other, may shift according to sex. If it is true that males are particularly vulnerable to pre-natal or very early life stressors, whereas females show special vulnerability at times of hormonal flux, this may also help in structuring prevention programs. Another moderating effect on psychiatric symptoms may turn out to be coping style. Strategies that use emotional processing and emotional expression to manage adversity may engender protective social support, but may do so more effectively in one sex than the other. Cognitive therapies may gain in effectiveness if they take sex into account.

There are many ways in which both sex and gender affect mental health. It is our belief that, by systematically including analyses of sex and/or gender in research, and conducting moderation analyses of sex/gender when interpreting study results, we can improve our understanding of differences between men and women's expression of disorder, and make sense of previously inconsistent study results. Our recommendation is to include sex and gender in the design phase of clinical trials in order to ensure sufficient power to conduct meaningful analyses. However, even in the absence of *a priori* inclusion of sex/gender, consideration of how results may potentially be affected by sex/gender would represent an improvement in trial methodology.

Improving the meaningfulness of psychiatric research goes beyond detecting group differences between men and women. When differences are found, it is important to search for underlying causes and to search for heterogeneity within the same sex.

This paper does not constitute a systematic search of the vast literature on all aspects of sex/gender effects on mental illnesses. Instead, it proposes a model that we hope readers will critique and expand on. One day, we will have a model that more completely accounts for the accumulating knowledge in this area of research. Our hope is that researchers across disciplines will test the theories presented and examine the intersection of sex, gender, and mental illness, thus continuing to contribute new insights that will advance the prevention of and recovery from mental illnesses.

## Data Availability Statement

The original contributions presented in the study are included in the article/supplementary material, further inquiries can be directed to the corresponding author/s.

## Author Contributions

DC, MM, and MS contributed to the article and approved of the final version. All authors contributed to the article and approved the submitted version.

## Conflict of Interest

The authors declare that the research was conducted in the absence of any commercial or financial relationships that could be construed as a potential conflict of interest.

## Publisher's Note

All claims expressed in this article are solely those of the authors and do not necessarily represent those of their affiliated organizations, or those of the publisher, the editors and the reviewers. Any product that may be evaluated in this article, or claim that may be made by its manufacturer, is not guaranteed or endorsed by the publisher.
